# Enlightening the toxinological dark matter of spider venom enzymes

**DOI:** 10.1038/s44185-024-00058-2

**Published:** 2024-09-13

**Authors:** Josephine Dresler, Volker Herzig, Andreas Vilcinskas, Tim Lüddecke

**Affiliations:** 1 Animal Venomics Lab, Department of Bioresources, Fraunhofer Institute for Molecular Biology and Applied Ecology, Gießen, Germany; 2 LOEWE Centre for Translational Biodiversity Genomics, Frankfurt a. M., Germany; 3 Centre for Bioinnovation, University of the Sunshine Coast, Sippy Downs, QLD Australia; 4 School of Science, Technology and Engineering, University of the Sunshine Coast, Sippy Downs, QLD Australia; 5 Institute for Insect Biotechnology, Justus-Liebig-University of Giessen, Gießen, Germany

**Keywords:** Enzymes, Chemical ecology, Animal physiology

## Abstract

Spiders produce highly adapted venoms featuring a complex mixture of biomolecules used mainly for hunting and defense. The most prominent components are peptidic neurotoxins, a major focus of research and drug development, whereas venom enzymes have been largely neglected. Nevertheless, investigation of venom enzymes not only reveals insights into their biological functions, but also provides templates for future industrial applications. Here we compared spider venom enzymes validated at protein level contained in the VenomZone database and from all publicly available proteo-transcriptomic spider venom datasets. We assigned reported enzymes to cellular processes and known venom functions, including toxicity, prey pre-digestion, venom preservation, venom component activation, and spreading factors. Our study unveiled extensive discrepancy between public databases and publications with regard to enzyme coverage, which impedes the development of novel spider venom enzyme-based applications. Uncovering the previously unrecognized abundance and diversity of venom enzymes will open new avenues for spider venom biodiscovery.

## Introduction

Spiders (Araneae) are predatory arthropods that inhabit virtually all terrestrial ecosystems^1^. During their >300 million years of evolutionary history, they evolved into highly effective predators of insects and other arthropods^2^. Despite the wide range of ecosystems conquered, their principal body plan has remained largely unchanged and is shared by all extant species across the three spider infraorders (Mesothelae, Mygalomorphae and Araneomorphae)^3–5^. The astonishing success of spiders in terms of biodiversity, evolutionary age and ecological versatility, is to a large extent rooted in key biomolecular innovations such as pheromone chemistry, silk production, and, in particular, venom^2,6–8^.

Venoms are complex chemical cocktails that evolved convergently multiple times in the animal kingdom^9^. They are actively injected from one animal into another, where they disturb vital physiological processes and cause damage or even death^10^. The bioactive components of venoms are described as toxins and are mostly of proteinaceous nature^10^. The three major biological functions of venom are hunting, defense and intraspecific competition, but additional minor functions include chemical communication and reproduction among others^11^.

Spiders are one of the oldest venomous lineages and also one of the most diverse, with ~51,000 described species^1^. This has led to the widespread recognition of spiders as the world’s most successful group of venomous animals, at least among terrestrial lineages^2^. The complexity of spider venom is also unmatched, featuring hundreds to thousands of different polypeptide components^12^. With all spiders except for the family Uloboridae being considered venomous^2^, spider venoms offer an immense library of bioactive components. Based on extrapolations from the limited number of species studied thus far, it has been estimated that up to 10 million biomolecules can be isolated from all extant species^13^.

Most known spider venom toxins are short disulfide-rich peptides with an inhibitor cysteine knot (ICK) motif^13^. These usually have molecular masses <7 kDa and are potent modulators of a wide range of ion channels. Their biological function is primarily trophic and secondarily defensive, and they are used to mount neurochemical attacks on insect prey or potential threats following injection via a cheliceral bite^2,14^. However, several of these ICK peptides also show promising activity against human ion channels involved in a wide range of neuropathologies. This bioactivity, paired with the extraordinary stability conferred by the ICK fold^15^, means that spider venom peptides are potent drug leads for the treatment of neurological diseases, including epilepsy, chronic pain and post-stroke brain damage^13,16–19^. Unsurprisingly, spider venom biodiscovery has focused on novel and potentially druggable ICKs and has typically involved bioactivity-guided screening, where crude venom is fractionated by chromatography and the fractions are analyzed by mass spectrometry to identify peptides with desired activities^20^. More recently, modern proteomics, transcriptomics and synthetic biology technologies were applied to venom systems and gave rise to a new field of research referred to as “venomics” (i.e. the application of omics and biotechnology to study venoms)^21,22^. Traditionally, it has been thought that spider venoms are predominantly composed of ICK peptides. Yet, a major insight from modern venomics is that they also contain multiple high-molecular-weight proteins, often with putative enzymatic activity and possible key functionality for the venom^2,23^.

Enzymes are complex proteins containing domains that catalyze chemical reactions. In general, the classification and nomenclature of enzymes depend on the catalyzed reaction and the substrates involved. In contrast to the frequent in-depth studies of neurotoxic peptides, the bioactivities of spider venom enzymes are almost entirely unknown. The only exceptions are sphingomyelinase D enzymes from sicariid spiders and a few neurotoxin-processing enzymes involved in toxin maturation^24–28^. However, in certain araneomorph spiders, enzymes may represent the main venom component^29,30^, which could indicate that they perform important biological functions that have yet to be fully understood. This growing body of evidence suggests that spider venoms contain a vast resource of undiscovered enzymes that can be thought of as toxinological dark matter. This is unfortunate because a better understanding of those molecules might provide further insights into the chemical and evolutionary ecology of spider venoms and could be employed as a valuable resource for translational research^31,32^. Hence, understanding and unveiling the diversity and biological significance of spider venom enzymes has recently been announced as one of the grand challenges in spider toxinology^33^.

Here we set out to provide the first holistic, structural assessment of the enzymatic diversity within spider venoms. Specifically, we analyzed the content of a manually curated venom database and mined putative enzymes from modern venomics research. We linked all the enzymes identified from databases and previously published venomics surveys with taxonomic classifications and potential functions. The wealth of data we analyzed shows that spider venoms are a rich source of enzymes and that the diversity of enzyme families rivals the number of known ICK families. Given the strong research bias towards ICK peptides and the recent advent of modern venomics, future studies should include the analysis of venom enzymes in more detail.

## Methods

### Database search

To investigate the diversity of known spider venom enzymes in public databases, we selected the VenomZone database as a source^34^. VenomZone is a publicly available database that is manually curated and contains entries of venom components representing six different lineages (snakes, spiders, cnidaria, insects, scorpions and cone snails) that are pre-categorized into distinct toxin families. Based on the pre-categorization we were able to manually screen all 1540 entries (accessed March 2023) of known spider venom components. This allowed us to retrieve all proteins assigned to known enzyme families. The entries including the original research paper in which they have been first described were collected in a master table, which is presented in Supplementary Table 1.

### Literature search

To identify spider venom enzymes other than those contained in the VenomZone database, we performed a literature search using Pubmed^35^. For this, we performed a total of five web searches using different combinations of the same keywords. Initially, we performed a search with the keywords “spider” and “venom” followed by three searches using both paired with one of the following keywords “venomics” or “transcriptomics” or “proteomics”, respectively. To ensure the holistic nature of our web search, we performed a fifth search using all terms simultaneously. This web search yielded >3700 peer-reviewed publications that we manually screened (both, main text and Supplementary Materials if provided) for spider venom enzymes. Identified hits were collected in a master table similarly to our data mining from databases (Supplementary Tables 2 and 3). Due to the assembly of artifacts, purely transcriptome-based studies tend to overestimate venom component diversity^36^. It is therefore best practice in modern venom research to only consider venom transcripts that have been validated at the protein level^36^. Thus, we opted to follow this conservative approach and only included enzymes that have been isolated or confirmed by mass spectrometry.

### Taxonomic classification

The taxonomy of spiders is in constant flux with hundreds of newly described species and genera each year and reoccurring modifications to the entire systematic system. As a consequence, generic and family-level assignments of spiders can tremendously change over time, especially in mygalomorph spiders which have dominated spider venom research. In order to reliably investigate the occurrence and abundance of venom components over larger taxonomic scales, it is pivotal to update retrieved venomic data to the most recent taxonomic classification. For this purpose, we utilized the World Spider Catalog (WSC), a taxonomic repository for spiders that is manually curated and maintains up-to-date taxonomic data for each known spider taxon across all levels of systematic hierarchy^1^. For each spider from which we identified enzymatic venom components in databases and/or web searches, we updated its current taxonomic status according to the most recent version of the WSC. Specifically, we curated genus and species names and paid special attention to correct family assignments, because these particularly affect conclusions drawn from larger phylogenetic analyses.

### Classification of the identified enzymes

To classify all identified enzymes, we employed the International Union of Biochemistry and Molecular Biology (IUBMB) enzyme classification. This uses a four-digit numerical system to classify enzymes based on Enzyme Commission (EC) numbers. The first number of this system encodes the type of reaction catalyzed. Until now, seven different enzyme classes are recognized: oxidoreductases (EC 1.-.-.-), transferases (EC 2.-.-.-), hydrolases (EC 3.-.-.-), lyases (EC 4.-.-.-), isomerases (EC 5.-.-.-), ligases (EC 6.-.-.-) and translocases (EC 7.-.-.-)^37^. The second digit represents the subclass, referring to the type of compound or group involved, while the third digit specifies the type of reaction involved in the sub-subclass. The fourth digit identifies each individual enzyme within the sub-subclass and represents it as a simple serial number^37^. Oxidoreductases (EC 1.-.-.-) for instance are subdivided into 26 subclasses, which are further classified into a total of 151 sub-subclasses. Each of these sub-subclasses contains individual enzymes which are identified according to a serial number^38^. As each provided EC number is unique if all digits are employed, the IUBMB system features a precise and simple means to classify and identify enzymes across the natural world.

For enzymes from databases specific EC identifiers usually have been provided and we have retrieved this information during data mining. However, for the enzymes identified during web searches in most cases no EC identifier has been provided. Consequently, we manually classified these following ENZYME^38^, a nomenclature database hosted by the Expasy bioinformatics portal of the Swiss Institute for Bioinformatics that describes each characterized enzyme type associated with an EC number. Following best practices in modern venomics experiments, full sequences and best BLAST results are usually provided for each identified component in the supplementary material and raw data of each publication. Whenever possible, we retrieved this information for each putative enzyme identified and used it to classify them following the EC system. Our cut-off criterion for classification was sequence similarity and we used the BLAST hit with the highest percentage of sequence similarity to classify presumed venom enzymes. Therefore, we first identified the EC identifier for the most similar BLAST hit for each identified enzyme and then assigned the respective compound the same complete EC number. Whenever no full sequences or sufficient annotation via BLAST searches were provided, we used the available information to assign as many digits of the EC system as possible. Here we retrieved the enzyme name provided by the main text of the manuscript or Supplementary Data and assigned EC identifiers based on protein names to the maximum level of certainty. For instance, all serine proteases that have not been specified in more detail were assigned to the general serine protease EC number 3.4.21.-. To maintain stringency no subgroup assignment was then performed.

To shed further light on the potential functional spectrum of the identified enzymes, we classified their biological functions within venom and the venom gland physiology. Since only a few spider venom enzymes have been studied so far, it is not possible to functionally characterize them based on experimental data. However, a recent study classified enzymes from scorpions based on thorough structural and functional analyses. Spiders and scorpions are closely related members of Arachnida whose venoms follow similar chemical trends and are employed for the same biological functions (i.e. hunting and defense). We therefore employed this functional classification of venom enzymes established for scorpions^39^ which distinguishes a total of seven global functions to scorpion venom enzymes: toxic, pre-digestion, spreading factor, precursor activation, preservative, multifunctional and undetermined. Accordingly to the classification established by Delgado-Prudencio et al.^39^, we then assigned a presumed biological function for each of the identified spider venom enzymes.

Besides biological functions within the venom, some spider enzymes have been recently classified for their potential cellular functions in the venom gland. This classification was implemented by Diniz et al.^40^ and applies gene ontology annotations of the cellular function category from known enzymes deposited in Uniprot which correspond to cellular components and roles in metabolism. Those relate to the production, maturation and secretion of complex toxin components including transcription, protein folding, transport and post-translational modifications, as well as ubiquitination and proteolysis^41^. We, therefore, employed the classification established by Diniz et al.^40^ to assign a cellular function for each putative spider venom enzyme. If the enzyme family has previously not been assigned a cellular function, we then refrained from annotating the cellular function. Since biological and cellular functions are not mutually exclusive, we assigned both when applicable. The combination of IUBMB, global venom function and cellular function classifications, allowed us to holistically interpret the identified spider venom enzymes for their chemical reactions and biological relevance in envenoming, venom gland physiology and beyond.

Another challenge besides the heterogeneous availability of classification data was the unclear enzymatic nature of some spider venom components. Prior works have identified some protein groups as potential enzymes, but those have not yet been verified for function, nor have they received any EC classification^42,43^. In order to represent as much molecular diversity as possible, we herein opted to also collect the distribution of those unclear components (e.g. CAP proteins and some lectins) but are aware that future works need to validate their activity, which might necessitate an update of the assessments provided in our study.

## Results

### VenomZone

VenomZone is a manually curated database of venom components containing 49 protein families, 17 of which are enzymatic. We found that eight known enzyme families occur in spider venoms, representing two distinct enzyme classes (hydrolases and isomerases). We also considered the potentially enzymatic glycoproteins. The enzymes identified in each class and the number of identified proteins for each family are discussed below and are summarized in Table 1.

**Table 1:** Diversity of spider venom enzymes present in the VenomZone database, showing assigned enzyme classes, families, EC numbers, taxonomic origins and number of proteins for each family

Enzyme class	Enzyme family	EC number	Spider family	No. of proteins
Hydrolase	Acetylcholinesterase	3.1.1.7	Barychelidae	1
	Phospholipase D	3.1.4.4	Sicariidae	218
	Hyaluronidase	3.2.1.-	Ctenidae Sicariidae	1 1
Isomerase	Reprolysin (M12B family)	3.4.24.-	Lycosidae	1
	Astacin (M12A family)	3.4.24.21	Sicariidae	5
	Peptidylprolyl isomerase	5.2.1.8	Agelenidae Ctenidae	2 1
Glycoproteins	CAP		Barychelidae Ctenidae Lycosidae	1 1 1
	Lectin		Ctenidae	1

A holistic overview of all components including corresponding references is provided in Supplementary Table 1.

Hydrolases use water to catalyze the cleavage of large proteins into smaller molecules^44^. Five distinct hydrolase families have been reported in spider venom. One acetylcholinesterase has been described in the Barychelidae (*Trittame*). The most diverse enzyme family is the arthropod dermonecrotic toxin phospholipase D, also known as sphingomyelinase D^25–28^ and we found 218 representing the family Sicariidae, specifically 174 from the genus *Loxosceles*, 22 from the genus *Hexophthalma* and 22 from the genus *Sicarius*. Furthermore, two hyaluronidases have been reported, one each from Ctenidae (*Phoneutria*) and Sicariidae (*Loxosceles*).

All the remaining hydrolases were metalloproteases. Metalloproteases are a group of proteases that require a metal which is involved in their catalytic mechanism^45^. We identified six members of the M12 family, comprising five astacins (M12A), all from Sicariidae (*Loxosceles*), and one partitagin (M12B, reprolysin) from Lycosidae (*Hippasa*).

Isomerases catalyze the structural conversion of molecules to their isomers^44^. We identified three peptidylprolyl isomerases, two from Agelenidae (*Agelena*) and one from Ctenidae (*Phoneutria*).

We identified four glycoproteins representing the cysteine-rich secretory proteins, antigen 5 and pathogenesis-related protein 1 (CAP) family as well as one lectin. The CAP family is associated with diverse functions. CAPs isolated from snake venom are neurotoxic whereas those from hematophagous species are potentially hemotoxic^10^, and little is known about spider venom CAPs. However, previous work suggested that based on sequence similarity to *Conus* CAP proteins they may represent a hitherto unrecognized lineage of proteases^29^ and we therefore included them in our survey. One CAP has been described thus far from each of the families Barychelidae (*Trittame*), Lycosidae (*Lycosa*) and Ctenidae (*Phoneutria*). The single lectin we identified was present in Ctenidae (*Phoneutria*). These carbohydrate-specific binding proteins that agglutinate cells or other materials are important for cell adhesion and defense^46,47^.

### Publications

To discover putative enzyme families that were previously overlooked, we collected all published spider venom proteo-transcriptomes from the last 19 years and analyzed them for the presence of enzymatic components. We identified 143 putative enzyme families that occur with varying numbers of identified proteins for each enzyme family in spider venoms (Table 2). The enzymes represent seven distinct classes and are described in more detail below.

**Table 2:** Diversity of spider venom enzymes present in publications, showing assigned enzyme classes, families, EC numbers and taxonomic origins

Enzyme class	Enzyme family	EC number	Spider family
Oxidoreductases	Synaptic vesicle membrane protein VAT-1 homolog-like	1.-.-.-	Theraphosidae
	Glucose 1-dehydrogenase	1.1.5.9	Eresidae
	3-Hydroxyisobutyrate dehydrogenase	1.1.1.31	Philodromidae
	Glucose-6-phosphate dehydrogenase	1.1.1.49	Theraphosidae
	Phosphoglycerate dehydrogenase	1.1.1.95	Lamponidae, Lycosidae, Theridiidae
	Glyceraldehyde-3-phosphate dehydrogenase	1.2.1.12	Eresidae, Theraphosidae
	Retinal dehydrogenase	1.2.1.36	Eresidae, Theraphosidae
	Acyl-CoA oxidase	1.3.3.6	Theraphosidae
	Glutamate dehydrogenase	1.4.1.3	Eresidae
	Uricase	1.7.3.3	Theraphosidae
	*γ*-Interferon-inducible lysosomal thiol reductase	1.8.-.-	Eresidae, Lamponidae, Theraphosidae
Transferases	Thiol oxidase	1.8.3.2	Theraphosidae
	Peroxidase	1.11.1.-	Lamponidae, Theraphosidae
	Glutathione peroxidase	1.11.1.9	Theraphosidae
	Thioredoxin-dependent peroxiredoxin	1.11.1.24	Theraphosidae
	Cysteine dioxygenase	1.13.11.20	Theraphosidae
	Procollagen-lysine 5-dioxygenase	1.14.11.4	Theraphosidae
	Dopamine *β*-monooxygenase	1.14.17.1	Ctenidae, Lamponidae, Theraphosidae
	Tyrosinase	1.14.18.1	Theraphosidae
	Superoxide dismutase	1.15.1.1	Ctenidae, Philodromidae, Theraphosidae
	Methyltransferase	2.1.1.-	Philodromidae, Theridiidae
	Trans-aconitate 2-methyltransferase	2.1.1.144	Philodromidae
	Type I protein arginine methyltransferase	2.1.1.319	Philodromidae
	Transketolase	2.2.1.1	Theraphosidae
	Transaldolase	2.2.1.2	Theraphosidae
	Arylamine *N*-acetyltransferase	2.3.1.5	Theraphosidae
	Phosphatidylcholine-sterol O-acyltransferase	2.3.1.43	Theraphosidae
	Glycylpeptide *N*-tetradecanoyltransferase	2.3.1.97	Theraphosidae
	cullin-RING-type E3 NEDD8 transferase	2.3.2.32	Philodromidae
	Polypeptide *N*-acetylgalactosaminyltransferase	2.4.1.41	Lamponidae
	Adenine phosphoribosyltransferase	2.4.2.7	Theraphosidae
	Spermidine synthase	2.5.1.16	Lamponidae, Theraphosidae
Hydrolases	Glutathione transferase	2.5.1.18	Eresidae, Theraphosidae
	Alanine transaminase	2.6.1.2	Philodromidae
	Kynurenine--oxoglutarate transaminase	2.6.1.7	Theraphosidae
	Protein kinase	2.7.-.-	Theraphosidae
	Glycerate 3-kinase	2.7.1.31	Theraphosidae
	Aminoglycoside 2″-phosphotransferase	2.7.1.190	Philodromidae
	Phosphoglycerate kinase	2.7.2.3	Philodromidae
	Arginine kinase	2.7.3.3	Linyphiidae, Lycosidae, Philodromidae, Theridiidae
	Nucleoside-diphosphate kinase	2.7.4.6	Lamponidae
	Non-specific serine/threonine protein kinase	2.7.11.1	Theraphosidae, Theridiidae
	[Mysosin light-chain] kinase	2.7.11.18	Theraphosidae
	Histidine kinase	2.7.13.3	Philodromidae, Theraphosidae
	Alcohol sulfotransferase	2.8.2.2	Eresidae
	Galactosylceramide sulfotransferase	2.8.2.11	Eresidae
	Nuclease (undetermined)	3.1.-.-	Theridiidae
	Phosphatases (undetermined)	3.1.-.-	Theridiidae
	Phospholipase (undetermined)	3.1.-.-	Theridiidae
	Lipase	3.1.1.-	Ctenidae, Lamponidae, Theraphosidae, Theridiidae
	Triacylglycerol lipase	3.1.1.3	Eresidae, Philodromidae, Tetragnathidae, Theraphosidae
	Phospholipase A2	3.1.1.4	Eresidae, Lamponidae, Linyphiidae, Pholcidae, Tetragnathidae, Theraphosidae, Trechaleidae
	Acetylcholinesterase	3.1.1.7	Barychelidae, Ctenidae, Lamponidae, Tetragnathidae, Theraphosidae
	Phospholipase A1	3.1.1.32	Theraphosidae
	S-formylglutathione hydrolase	3.1.2.12	Theraphosidae
	Palmitoyl-protein hydrolase	3.1.2.22	Theraphosidae
	Alkaline Phosphatase	3.1.3.1	Theridiidae
	5′ Nucleotidase	3.1.3.5	Araneidae, Eresidae, Theraphosidae
	Phosphoglycolate phosphatase	3.1.3.18	Philodromidae
	Protein tyrosine phosphatase	3.1.3.48	Lamponidae, Tetragnathidae, Theraphosidae, Theridiidae
	Phosphatidylinositol-3,4-bisphosphate 4-phosphatase	3.1.3.66	Araneidae
	Phospholipase D	3.1.4.4	Sicariidae, Theraphosidae
	Sphingomyelin phosphodiesterase	3.1.4.12	Sicariidae
	*N*-acetylgalactosamine-6-sulfatase	3.1.6.4	Theraphosidae
	Deoxyribonuclease 1-like 3	3.1.21.-	Theridiidae
	Hyaluronidase (glycosyl hydrolase 56)	3.2.1.-	Atracidae, Ctenidae, Lamponidae, Lycosidae, Tetragnathidae, Theraphosidae, Theridiidae, Trechaleidae
	Neutral alpha-glucosidase (glycosyl hydrolase 31)	3.2.1.-	Lamponidae
	Glycosyl hydrolase	3.2.1.-	Theraphosidae
	*α*-Amylase	3.2.1.1	Eresidae, Tetragnathidae, Theridiidae, Trechaleidae
	Chitinase	3.2.1.14	Araneidae, Ctenidae, Eresidae, Lamponidae, Pholcidae, Tetragnathidae, Theridiidae
	Lysozyme	3.2.1.17	Araneidae, Theridiidae
	*α*-Galactosidase	3.2.1.22	Theraphosidae
	*β*-Galactosidase	3.2.1.23	Theraphosidae
	*α*-Mannosidase	3.2.1.24	Eresidae, Theridiidae
	Hexosaminidase (glycosyl hydrolase 20)	3.2.1.52	Lamponidae, Theraphosidae, Theridiidae
	8-Oxoguanine DNA glycosylase	3.2.2.-	Theraphosidae
	Cathepsin	3.4.-.-	Ctenidae, Lamponidae, Theraphosidae, Theridiidae
	SPase	3.4.-.-	Trechaleidae
Lyases	Metalloproteinase (undetermined)	3.4.-.-	Ctenidae, Theraphosidae
	Peptidase (undetermined)	3.4.-.-	Theraphosidae, Theridiidae
	Phage endopeptidase	3.4.-.-	Theraphosidae
	Aminopeptidase (undetermined)	3.4.11.-	Eresidae, Theraphosidae
	Aminopeptidase O	3.4.11.-	Philodromidae
	Membrane alanyl aminopeptidase	3.4.11.2	Eresidae, Theraphosidae
	Cytosol non-specific dipeptidase	3.4.13.18	Theraphosidae
	Dipeptidyl-peptidase I	3.4.14.1	Theraphosidae
	Angiotensin-converting enzyme	3.4.15.1	Ctenidae, Eresidae, Lamponidae, Linyphiidae, Lycosidae, Theraphosidae, Theridiidae, Trechaleidae
	Carboxypeptidase (undetermined)	3.4.16.-	Theridiidae
	Carboxypeptidase C	3.4.16.5	Araneidae, Lamponidae
	Carboxypeptidase D	3.4.16.6	Eresidae, Theraphosidae, Theridiidae
	Metallocarboxypeptidase (undetermined)	3.4.17.-	Theraphosidae
	Carboxypeptidase A	3.4.17.1	Theraphosidae, Trechaleidae
	Carboxypeptidase B	3.4.17.2	Eresidae, Theridiidae
	Carboxypeptidase E	3.4.17.10	Theraphosidae
	Carboxypeptidase M	3.4.17.12	Tetragnathidae
	Folate gamma-glutamyl hydrolase	3.4.19.9	Theraphosidae
	S1 Protease	3.4.21.-	Araneidae, Ctenidae, Eresidae, Lycosidae, Philodromidae, Pholcidae, Theraphosidae, Theridiidae, Trechaleidae
	Trypsin	3.4.21.4	Lamponidae, Theraphosidae, Theridiidae
	Coagulation Factor Xa	3.4.21.6	Theraphosidae
	Proprotein convertase 1	3.4.21.93	Eresidae, Lamponidae, Pholcidae, Tetragnathidae, Theraphosidae, Theridiidae
	Equine arterivirus serine peptidase	3.4.21.114	Philodromidae
	Cathepsin B	3.4.22.1	Theraphosidae
	Cathepsin L	3.4.22.15	Eresidae, Theridiidae
	legumain	3.4.22.34	Theraphosidae
	Bleomycin hydrolase	3.4.22.40	Araneidae
	Lysosomal aspartic protease	3.4.23.-	Lamponidae
	Metalloendopeptidase (undetermined)	3.4.24.-	Ctenidae, Eresidae, Philodromidae, Theraphosidae, Theridiidae
	Neprilysin	3.4.24.11	Atracidae, Barychelidae, Eresidae, Lamponidae, Pholcidae, Tetragnathidae, Theraphosidae, Theridiidae
	Astacin (M12A family)	3.4.24.21	Araneidae, Ctenidae, Eresidae, Plectreuridae, Pholcidae, Theridiidae
	Endothelin-converting enzyme	3.4.24.71	Eresidae, Lamponidae, Theridiidae
	Proteasome endopeptidase complex	3.4.25.1	Theridiidae
	Arylformamidase	3.5.1.9	Philodromidae, Theraphosidae
	Ceramidase	3.5.1.23	Lamponidae, Theraphosidae
	Pantetheine hydrolase	3.5.1.92	Eresidae, Theraphosidae
	Dihydropyrimidinase	3.5.2.2	Theraphosidae
	2-Iminobutanoate/2-iminopropanoate deaminase	3.5.99.10	Philodromidae
	Helicase	3.6.4.-	Lamponidae
	Aromatic l-amino-acid decarboxylase	4.1.1.28	Araneidae
	Carbonic anhydrase	4.2.1.1	Lamponidae, Theraphosidae
	Enolase	4.2.1.11	Lamponidae
	Aconitate hydrolase	4.2.1.3	Philodromidae
	Dermonecrotic protein 1 LiDl	4.6.1.-	Sicariidae
	Peptidylprolyl isomerase	5.2.1.8	Ctenidae, Lamponidae, Philodromidae
Isomerases	Triose-phosphate isomerase	5.3.1.1	Theridiidae
	Protein disulfide isomerase	5.3.4.1	Eresidae, Philodromidae, Theraphosidae, Trechaleidae
	Phosphomannomutase	5.4.2.8	Theraphosidae
	Ligase	6.-.-.-	Theraphosidae
Ligases	Acyl-CoA synthetase	6.2.1.3	Araneidae
	Carbamoyl-phosphate synthase	6.3.4.16	Theraphosidae
Translocases	ATP synthase	7.1.2.2	Eresidae, Lamponidae, Philodromidae, Theraphosidae, Theridiidae
	Ca^2+^-transporting ATPase	7.2.2.10	Theridiidae
Glycoproteins	CAP		Atracidae, Araneidae, Barychelidae, Ctenidae, Linyphiidae, Lycosidae, Philodromidae, Pholcidae, Tetragnathidae, Theraphosidae, Theridiidae, Trechaleidae
	Lectin		Ctenidae, Lamponidae, Theraphosidae
Superfamily	*α*/*β* Hydrolase superfamily	1.-.-.- –6.-.-.-	Theraphosidae
	Peptidylglycine monooxygenase	1.14.17.3	Eresidae, Theraphosidae, Theridiidae, Trechaleidae
	*α*-Aminoadipic semialdehyde synthase	1.5.1.8/ 1.5.1.9	Philodromidae
Multidomain enzymes	Histone-lysine *N*-methyltransferase ASH1L	2.1.1.359/ 2.1.1.367	Theraphosidae
	Cyclin-dependent kinase 9	2.7.11.22/ 2.7.11.23	Philodromidae
	Dual-specificity phosphatase 23	3.1.3.16/ 3.1.3.48	Theraphosidae

A holistic overview of all components including corresponding references is provided in Supplementary Tables 2 and 3.

Oxidoreductases facilitate the reduction and oxidation of molecules through the transfer of electrons^44^. We identified 52 oxidoreductases belonging to 20 enzyme families, many from the spider family Theraphosidae. These include the synaptic vesicle membrane protein VAT-1 homolog-like, a thiol oxidase, two cysteine dioxygenases and two glucose-6-phosphate dehydrogenases in the genus *Acanthoscurria*, as well as 13 acyl-CoA oxidases and one uricase in *Chilobrachys guangxiensissis*. Furthermore, five phosphoglycerate dehydrogenases have been described, one from Lamponidae (genus *Lampona*), one from Lycosidae (*Lycosa tarantula)*, and the remaining three from Theridiidae (genus *Latrodectus*). A *γ*-interferon-inducible lysosomal thiol reductase has been described in Lamponidae (*Lampona*), one in Theraphosidae (*Acanthoscurria*) and one in Eresidae (*Stegodyphus*). A peroxidase has been described in the family Lamponidae (*Lampona*) and one in the family Eresidae (*Stegodyphus*) and dopamine *β*-hydroxylases have been identified in three spider families, one each in Ctenidae (*Phoneutria*), Lamponidae (*Lampona*) and Theraphosidae (*Acanthoscurria*). One tyrosinase was identified in the theraphosid spider *Cyriopagopus schmidti* (*C. schmidti*). Finally, a superoxide dismutase has been described twice in the family Ctenidae (*Phoneutria*), once in the family Philodromidae (*Tibellus*) and twice in Theraphosidae (*Acanthoscurria*). Three retinal dehydrogenases have been reported in Theraphosidae (*Acanthoscurria*) and one in Eresidae (*Stegodyphus*). In the family Eresidae one glucose 1-dehydrogenase, one glutamate dehydrogenase and one glutathione peroxidase has been reported. A glyceraldehyde-3-phosphate dehydrogenase has been described in Theraphosidae (*Acanthoscurria*) and in Eresidae (*Stegodyphus*). The latter also features a 3-hydroxyisobutyrate dehydrogenase, a procollagen-lysine 5-dioxygenase and two thioredoxin-dependent peroxiredoxins.

Transferases catalyze the transfer of specific functional groups between molecules^44^. We identified 49 transferases belonging to 27 enzyme families, spanning a range of diverse activities. Three methyltransferases have been described, one from the family Philodromidae (*Tibellus*) and two from Theridiidae (*Latrodectus)*. The genus *Tibellus* also features one cullin-RING-type E3 NEDD8 transferase, one alanine transaminase, one aminoglycoside 2″-phosphotransferase, one phosphoglycerate kinase, one trans-aconitate 2-methyltransferase and one type I protein arginine methyltransferase. Furthermore, two transketolase, one transaldolase, one arylamine *N*-acetyltransferase, one glycylpeptide *N*-tetradecanoyltransferase and one adenine phosphoribosyltransferase were identified in spiders from the family Theraphosidae. One polypeptide *N*-acetylgalactosaminyltransferase has been found in Lamponidae (*Lampona*) along with a spermidine synthase. Three further spermidine synthases have been found in Theraphosidae (*Acanthoscurria*) along with three glutathione transferases, two from Theraphosidae (*Chilobrachys*) and one from Eresidae (*Stegodyphus*). Additionally, one kynurenine-oxoglutarate transaminase, one protein kinase and one glycerate 3-kinase were reported in the theraphosid spider *C. schmidti*. Nine arginine kinases have been reported, two from Linyphiidae (*Hylyphantes*), one from Lycosidae (*Lycosa*), one from Philodromidae (*Tibellus*) and five from Theridiidae (*Latrodectus*). One nucleoside-diphosphate kinase has been described in Lamponidae (*Lampona*) and six non-specific serine/threonine protein kinases have been identified, four in the family Theraphosidae (two in *Cyriopagopus*, one in *Pamphobeteus* and one in *Acanthoscurria*) and two in Theridiidae (*Latrodectus*). One galactosylceramide sulfotransferase and one alcohol sulfotransferase have been reported from Eresidae (*Stegodyphus*) and one phosphatidylcholine-sterol O-acyltransferase from Theraphosidae (*Acanthoscurria*). Finally, a myosin light-chain kinase has been identified twice in Theraphosidae (*Chilobrachys*) and a histidine kinase has been found in Philodromidae (*Tibellus*) and Theraphosidae (*Chilobrachys)*.

The most diverse enzyme class we identified in spider venoms are the hydrolases, with 516 hydrolases belonging to 74 different enzyme families, comprising 21 acting on ester bonds, 12 glycosylases, 35 peptidases, five acting on carbon–nitrogen bonds and one acting on acid anhydrides, which are discussed below.

The group acting on ester bonds includes three undetermined enzymes in the family Theridiidae, three undetermined nucleases, five undetermined phosphatases and one undetermined phospholipase. Others have been assigned more specifically. For example, two lipases have been reported in Ctenidae (*Phoneutria*), one each in Lamponidae (*Lampona*), Theraphosidae (*Acanthoscurria)*, and Theridiidae (*Parasteatoda*), and an unreported number of additional lipases has been found in Theridiidae (*Steatoda*). The distribution of triacylglycerol lipases, phospholipases, acetylcholinesterases and other ester-specific hydrolases is summarized in Supplementary Table 3.

Among the glycosylases, we found *α*-amylases in Tetragnathidae (*Tetragnatha*), Theridiidae, Trechaleidae (*Cupiennius*) and Eresidae (*Stegodyphus*), as well as 25 hyaluronidases distributed among eight spider families. We also found one neutral *α*-glucosidase (Lamponidae), one glycosyl hydrolase (Theraphosidae) and 19 chitinases in seven families. Two lysozymes were identified, one each in Araneidae (*Araneus*) and Theridiidae (*Latrodectus*). We also found a *α*-galactosidase and two *β*-galactosidases in Theraphosidae (*Acanthoscurria*) and three *α*-mannosidases, two in Theridiidae (*Parasteatoda*) and one in the Eresidae (*Stegodyphus*). The distribution of these enzymes and other glycosylates is summarized in Supplementary Table 3.

The 35 peptidases we identified included cathepsins in 5 spider families and metalloproteases in Ctenidae (*Phoneutria*) and Theraphosidae (*Acanthoscurria*). Twenty-one undetermined peptidases were described in the family Theraphosidae (14 in *Acanthoscurria*) and Theridiidae (seven in *Latrodectus*). We identified ten angiotensin-converting enzymes in eight spider families. Carboxypeptidases were also widely represented, including two of the carboxypeptidase C family in Araneidae (*Argiope*) and Lamponidae (*Lampona*), one carboxypeptidase D each in Theridiidae (*Parasteatoda)*, Eresidae (*Stegodyphus*) and Theraphosidae (*Acanthoscurria*), three of the carboxypeptidase A family two in Theraphosidae (*Acanthoscurria*) and one in Trechaleidae (*Cupiennius*), two carboxypeptidases B from Theridiidae (*Parasteatoda*) and Eresidae (*Stegodyphus*), one carboxypeptidase E from Theraphosidae (*Acanthoscurria*) and two carboxypeptidases M from Tetragnathidae (*Tetragnatha*). We found 19 S1 proteases in 10 spider families, and 8 proprotein convertases in 6 spider families. Neprilysin was represented 46 times in 8 eight spider families, 17 from Pholcidae (*Physocyclus*). We found 30 astacins in five families and an undefined number of further examples in Theridiidae (*Steatoda*). The distribution of these enzymes and other glycosylates is summarized in Supplementary Table 3.

The five enzyme families specific for carbon–nitrogen bonds comprised an arylformamidase identified twice in Philodromidae (*Tibellus*) and once in Theraphosidae (*Cyriopagopus*), a ceramidase found once each in Lamponidae (*Lampona*) and Theraphosidae (*Acanthoscurria*), a pantetheine hydrolase in Theraphosidae (*Acanthoscurria*) and Eresidae (*Stegodyphus*), two dihydropyrimidinases from Theraphosidae (*Chilobrachys*) and one 2-iminobutanoate/2-iminopropanoate deaminase from Philodromidae (*Tibellus*). The last of the hydrolases is the helicase which acts on acid anhydrides and has been detected in the Lamponidae (*Lampona*).

Lyases facilitate the cleavage of several chemical bonds other than hydrolysis or oxidation^37,44^. We identified 12 lyases belonging to 5 enzyme families. These comprised one aromatic l-amino-acid decarboxylase in Araneidae (*Araneus*) and eight carbonic anhydrases, two in Lamponidae (*Lampona*) and six in the Theraphosidae (*Acanthoscurria*), as well as one enolase in Lamponidae (*Lampona*), one aconitate hydrolase in Philodromidae (*Tibellus*) and one dermonecrotic protein 1 LiDl in Sicariidae (*Loxosceles*).

We identified 15 isomerases in 4 enzyme families. Six peptidyl-prolyl *cis*–*trans* isomerases have been reported, two each in the families Lamponidae (*Lampona*), Philodromidae (*Tibellus*) and Ctenidae (*Phoneutria*). Two triose-phosphate isomerases have been described from Theridiidae (*Latrodectus*) as well as six protein disulfide isomerases, one from Trechaleidae (*Cupiennius*), three from Philodromidae (*Tibellus*), one from Eresidae (*Stegodyphus*) and one from Theraphosidae (*Acanthoscurria*). Finally, a single phosphomannomutase has been identified in the theraphosid spider *C. schmidti*.

Ligases represent an enzyme class that catalyzes the formation of chemical bonds between two large molecules, usually involving ATP or other high-energy donors^44^. We identified three ligases belonging to three enzyme families. One of them could only be assigned to the first number of the four-digit EC system (EC 6.-.-.-). This ligase has been reported from Theraphosidae (*Cyriopagopus*). For the remaining ligases, we were able to identify all four digits of the EC system, describing one acyl-CoA synthetase from Araneidae (*Araneus)*, and one carbamoyl-phosphate synthase from Theraphosidae (*Cyriopagopus*).

Translocases catalyze the transport of ions or molecules across membranes^37^. We identified 40 translocases belonging to two enzyme families. This includes 30 ATP synthases that have been reported in five spider families. One is from the family Lamponidae (*Lampona*), six from Philodromidae (*Tibellus*), two from Theraphosidae (one each from *Chilobrachys* and *Cyriopagopus*), 19 from Theridiidae (*Latrodectus*) and one from Eresidae (*Stegodyphus*). Furthermore, ten Ca^2+^-transporting ATPases have been identified in the theridiid spider *Latrodectus tredecimguttatus*.

Multidomain enzymes described enzymes which are assigned to one EC number but contain multiple enzymatically active domains. We identified 13 multidomain enzymes belonging to five enzyme families. For example, peptidylglycine monooxygenases contain two separate catalytic domains, but the enzyme is assigned only one EC number^48^. Eight have been described in four spider families, four in Theraphosidae (*Acanthoscurria*), one in Theridiidae (*Parasteatoda*), one in Trechaleidae (*Cupiennius*), two in Eresidae (*Stegadyphus*) and more in Theridiidae (*Steadoa*) but the precise number has not been reported. One *α*-aminoadipic semialdehyde synthase has been detected in Philodromidae (*Tibellus)* and one histone-lysine *N*-methyltransferase ASH1L from Theraphosidae (*Acanthoscurria*). Two cyclin-dependent kinases have been found in Philodromidae (*Tibellus*) and one dual-specificity phosphatase 23 in Theraphosidae (*Cyriopagopus*). In contrast to the peptidylglycine monooxygenase, these enzymes have more than one EC number, each representing a different enzymatically active domain.

We identified 78 glycoproteins belonging to two enzyme families, i.e. 73 CAPs and five lectins distributed throughout the spider phylogenetic tree. Three CAPs were reported in Atracidae (*Hadronyche*), 26 in Araneidae (12 *Argiope* and 14 *Araneus*), one in Barychelidae (*Trittame*), five in Ctenidae (*Phoneutria*), three in Linyphiidae (*Hylyphantes*), four in Lycosidae (*Lycosa*), two in Philodromidae (*Tibellus*), two in Pholcidae (*Physocyclus*), two in Tetragnathidae (*Tetragnatha*), 14 in Theraphosidae (nine in *Acanthoscurria*, five in *Pamphobeteus*), nine in Theridiidae (one in *Parasteatoda*, eight in *Steatoda*) and two in Trechaleidae (*Cupiennius*). Lectin has been found once in the families Ctenidae (*Phoneutria*) and Lamponidae (*Lampona*) and twice in Theraphosidae (*Acanthoscurria*).

One superfamily containing *α*/*β*-hydrolases has been detected in spider venom. One member of this superfamily has been identified in Theraphosidae (*Cyriopagopus*) but given that it contains enzymes representing enzyme classes EC 1.-.-.- –6.-.-.-, no further classification was possible.

### Discrepancies between database entries and venomics studies

A comparison of enzymes deposited in databases and those mined from venomic data revealed extensive discrepancies. Only eight enzyme families are described in VenomZone, whereas we identified 143 enzyme families by screening published venomic data. Seven of these enzyme families were found in both sources, revealing that 136 enzyme families identified in the literature are not found in the VenomZone database. Only the partitagins (M12B family) are exclusively found in VenomZone, and even this family may be included among the many undetermined metalloproteases described in the literature that are not specifically identified in the proteo-transcriptomics data.

### Biological function of the enzymes

**Fig. 1:**
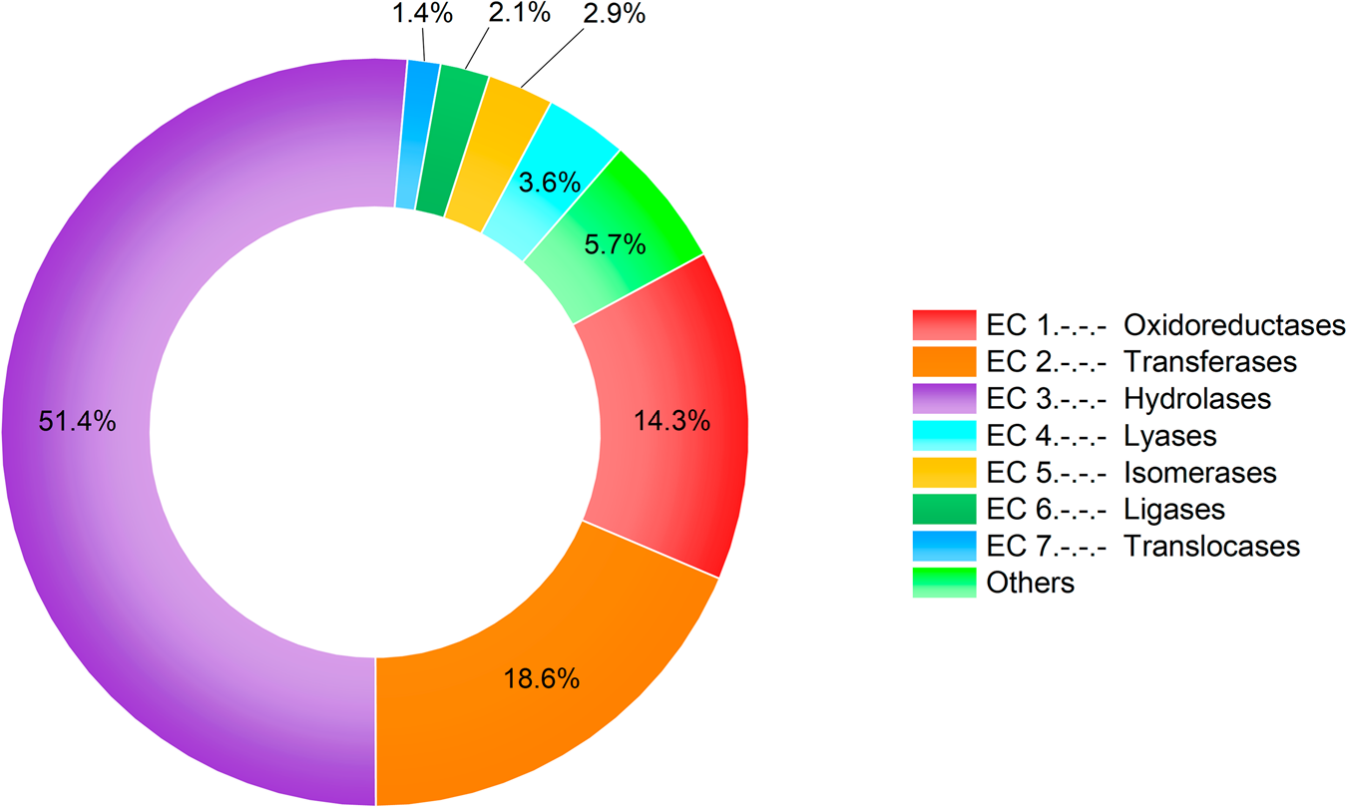
The diversity of spider venom enzymes. Relative distribution of spider venom enzyme families from VenomZone entries and proteo-transcriptomic studies per enzyme class. The category “others” includes glycoproteins, multidomain enzymes and the superfamily (α/β-hydrolases).

Spiders seem to utilize all distinct enzyme classes with the majority (84.3%) being hydrolases (51.4%), transferases (18.6%) and oxidoreductases (14.3%) (Fig. 1). To develop a deeper understanding of the identified enzymes we sought to assign putative venom functions using the functional classifications.

**Fig. 2:**
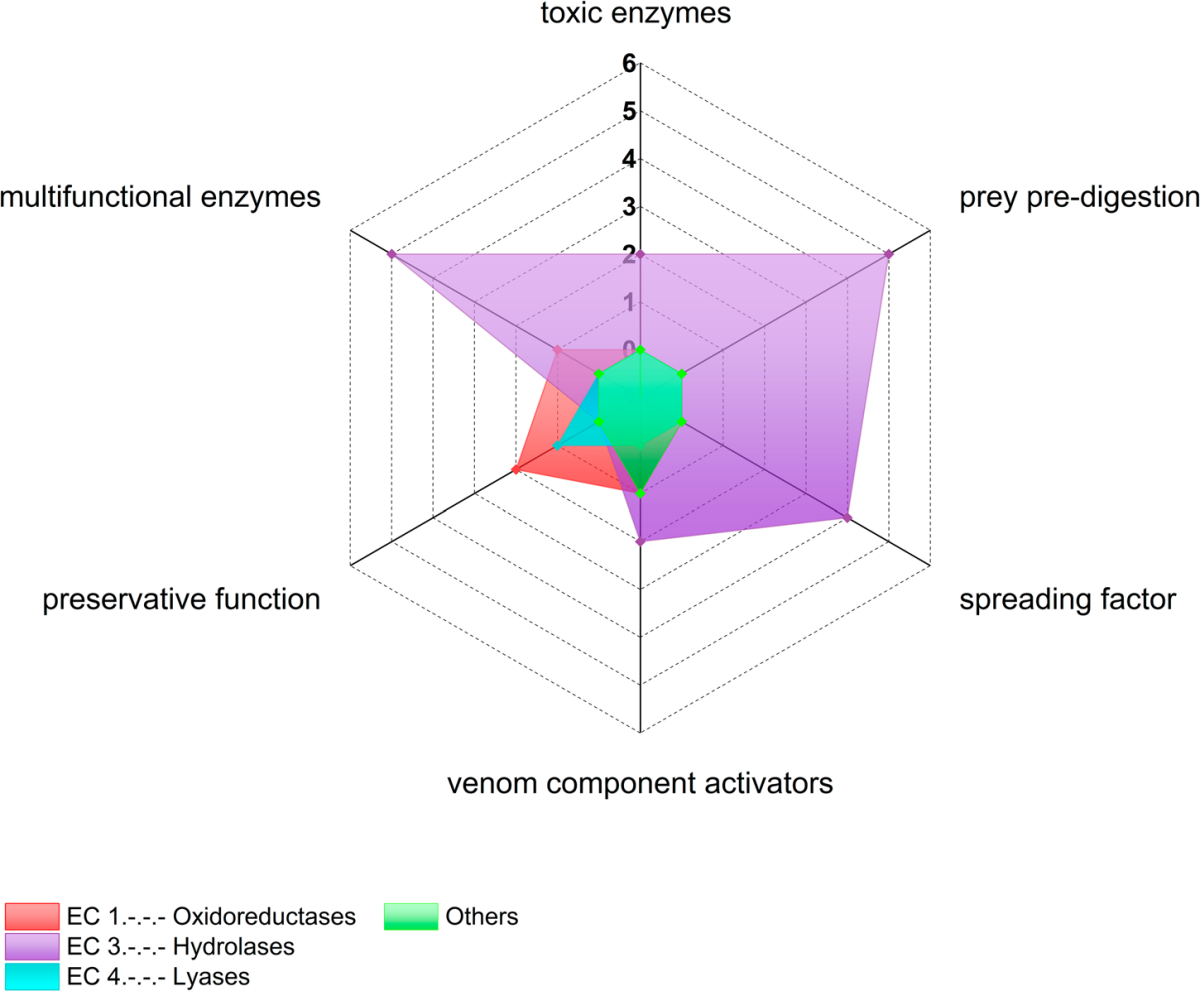
The spectrum of biological functions of spider venom enzymes. The radar plot shows the number of identified spider venom enzyme classes for each of the previously established functional classes of arachnid venom enzymes. The category “others” includes glycoproteins, multidomain enzymes and the superfamily (α/β-hydrolases). Counts show the number of enzymes per category.

According to our criteria, we were able to assign 24 of the 144 enzymes to physiological functions. Members of the phospholipase D and acetylcholinesterase families were assigned as toxic enzymes, whereas triacylglycerol lipases, chitinases, *α*-amylases, *α*-galactosidases and ceramidases are thought to be involved in the pre-digestion of prey. Furthermore, 5′ nucleotidases, hyaluronidases, angiotensin-converting enzymes and coagulation factor Xa may act as spreading factors, whereas dopamine *β*-monooxygenase, lysozymes, carboxypeptidases E and peptidylglycine monooxygenases are thought to activate venom components. Peroxidases, superoxide dismutases and carbonic anhydrases putatively have preservative functions whereas thioredoxin-dependent peroxiredoxins, phospholipases A2, carboxypeptidases B, trypsin, neprilysin and astacin have multiple enzymatic functions in venom (Supplementary Table 4). Figure 2 shows enzyme classes linked to physiological functions. Despite the remarkable abundance of hydrolases, only some oxidoreductases and one lyase seem to have known associated venom function. Hydrolases play a role in every function of venom except preservation. Interestingly, the functions assigned to oxidoreductases and the one lyase include preservation, with oxidoreductases also activating venom components.

We also assigned 17 of the enzymes to cellular functions such as metabolism or a contribution to cellular components^40^. Five of them are oxidoreductases (glucose-6-phosphate dehydrogenase, glucose 1-dehydrogenase, *γ*-interferon-inducible lysosomal thiol reductase, and dopamine *β*-monooxygenase, glutathione peroxidase), two are transferases (arginine kinase and nucleoside-diphosphate kinase), five are hydrolases (*α*-mannosidase, hexosaminidase, proprotein convertase 1, S1 proteases and metalloendopeptidases), one is a lyase (carbonic anhydrase), two are isomerases (peptidyl-prolyl isomerase and protein disulfide isomerase), one is the multidomain enzyme peptidylglycine monooxygenase and lectins (Supplementary Table 4).

## Discussion

In this study, we have provided the first systematic analysis of the distribution, abundance and biological significance of spider venom enzymes. Our results show many different enzymes spread across the spider tree of life. Overall, 144 enzyme families have been described from 17 spider families, eight in the VenomZone database whereas 136 are exclusively found in proteo-transcriptome data. These are distributed in all enzyme classes, which highlights the chemical diversity of spider venoms (Fig. 1). Neurotoxins are well-known as the major component of spider venom whereas enzymes are considered to be minor components^24–28^. Because of this, and given their potential as drug leads, neurotoxins have been the focus of many studies, whereas enzymes have been mostly overlooked. We found that venom enzymes show remarkable diversity, comparable to that of the neurotoxins. In some cases, the number of sequences identified in the proteome data was also comparably high. For example, *Phoneutria nigriventer* venom proteome consists of ~42% neurotoxins and ~43% enzymes^40^ whereas the venom of *Steatoda nobilis* only features ~15% enzymes compared to ~49% neurotoxins, while the remaining proteins possess other functions or unknown biological activities^49^. The abundance of the identified spider venom enzyme sequences therefore seems to vary depending on the species and further adds to the overall diversity of spider venom composition.

**Fig. 3:**
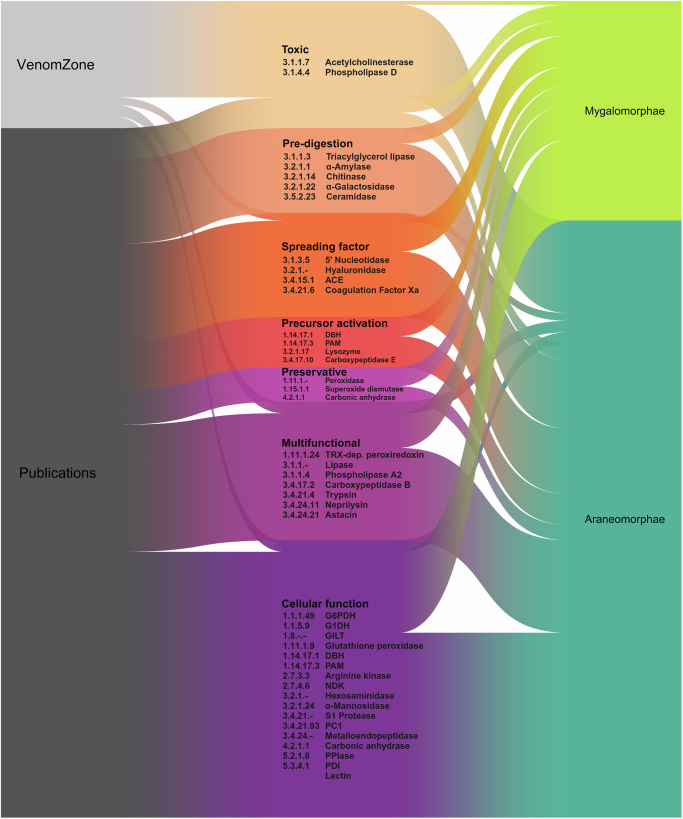
Identified spider venom enzymes in light of biological function and taxonomic origin. Sankey plot showing the relationship between venom enzymes identified from the VenomZone database and publications (left side), their associated functions (middle part) and presence in spider infraorders (right side). ACE angiotensin-converting enzyme, DBH dopamine *β*-monooxygenase, PAM peptidylglycine monooxygenase, TRX-dep. peroxiredoxin thioredoxin-dependent peroxiredoxin, G6PDH glucose-6-phosphate dehydrogenase, G1DH glucose 1-dehydrogenase, GILT *γ*-interferon-inducible lysosomal thiol reductase, NDK nucleoside-diphosphate kinase, PC1 proprotein convertase 1, PPIase peptidylprolyl isomerase, PDI protein disulfide isomerase.

Spiders are mainly investigated if they are large, like many of the mygalomorphs^19,20^, or if they are medically relevant in humans, such species in the genera *Loxosceles* or *Latrodectus*^50^. Considering the number of enzymes described throughout the phylogenetic tree, we see the greatest abundance in modern spiders from the infraorder Araneomorphae (Araneoidea and RTA clade) and in the more primitive family Theraphosidae which is part of the Mygalomorphae (Fig. 3). This is supported by the number proteo-transcriptomic studies investigating theraphosid (eight), theridiid (five), araneid (three) and ctenid (three) venom. Theraphosid spiders are among the largest spiders, explaining the increased attention they receive from the scientific community. A large proportion of reported toxins are derived from mygalomorph species^51^, while other spider families have been largely overlooked in venom research^50^. However, we found that the situation is currently undergoing a change with recent publications focusing on four previously neglected spider families^52^.

Our study revealed a large difference between databases and publicly available data. We found that the number of identified spider venom enzymes increased in the recent past, including many small araneomorph species, but these data have apparently not yet found their way into the respective databases. The traditional venom characterization pipeline involves bioactivity screening of crude venoms, which is dependent on the often limited venom yield of the chosen source species^51^. More recently, the characterization of venom has been facilitated by omics technologies particularly transcriptomics and proteomics. These combine bioinformatics tools with existing functional annotations of known toxins from databases to identify proteinaceous components and predict their function from minuscule starting material^51,53–55^. This enables the examination of venoms that were traditionally not accessible^51^. The apparent absence of most spider venom enzymes from public databases may therefore reflect the dominance of components identified using the less sensitive methodologies, the restricted range of target species previously examined, and the persistent focus on small neurotoxic peptides from spider venoms. Another explanation could be that public databases have not yet caught up with more recent discoveries in spider venom enzymes.

Our analysis revealed that although many spider venom enzymes have already been discovered, only a small fraction has yet been incorporated into public databases. However, providing easy public access to spider venom enzymes is crucial for facilitating our understanding of spider venom ecology, biochemistry and physiology, and it also underpins some important considerations for spider venom biodiscovery strategies as a whole.

The widespread neglect of enzymatic components in public databases has potential detrimental effects for future biodiscovery programs. In a venomics experiment, lineage-specific databases are the principal means to functionally annotate amino-acid sequences encoding for venom components. If suitable database entries for a given protein class are not present in the chosen database, then functional annotations will not be feasible. As for spiders, the ArachnoServer database has been the repository of choice for the annotation within spider venomics experiments. However, due to an ongoing major refurbishment, this important resource is currently being reduced to a temporary version with only limited functionality^33^. Since, VenomZone has emerged as the a priori repository and is accordingly the most utilized database in this field. Our analysis shows that the vast majority of enzyme families that have been identified from spider venoms are absent from the VenomZone database. Thus, if only VenomZone is used for venomics experiments, the conducted venom profiling will be blind towards the missing components. Therefore, the absence of spider venom enzymes from databases bears the risk of resulting in incomplete venom profiling that lacks important biological and translational details. Given the pivotal importance of databases, the establishment of a global arachnid venom repository has been recognized as an urgently needed resource, with the new version of ArachnoServer being planned to also include data on venom enzymes^33^. Based on our findings, we recommend that all arachnid venom repositories need to include data on spider venom enzymes to better reflect the entire molecular diversity within arachnid venoms and to remove a persistent blind spot in the field of spider toxinology. This might not only lead to important discoveries revolving around spider venom biology, but also yield a plethora of useful enzymes for biotechnological applications.

Considering the apparent diversity of spider venom enzymes, the question may arise as to why they have been absent from databases so persistently? For this, a variety of potential explanations come to mind that deserve discussion. The most obvious may be the strong anthropocentric bias towards biomedicine. Most studies have been carried out by isolating peptides with desired activities and therefore other components, including enzymes have been neglected. Accordingly, they were rarely characterized and introduced to databases. In recent works, a larger diversity of hitherto neglected components was reported, but still only rarely included to public repositories. This may be linked to the tendency of venomics studies to not always provide all sequence data to desirably extents. Quite commonly, neither transcriptomic nor proteomic raw data is uploaded to archives such as PRIDE or SRA. Likewise, multiple studies missed the opportunity to provide all relevant information upon identified venom components in their article (or supplements). For including identified proteins into public databases (Uniprot, VenomZone, etc.), these components usually have to be submitted actively by the responsible researcher, which has not yet received the desired priority. As a result, at least a larger fraction of the discrepancy between database entries and literature-derived spider venom enzymes may be explained. This leads us to propose that the spider toxinology community needs to improve on this end and should aim to publish raw data and submit identified components to repositories more actively.

Spiders use their venoms mainly for hunting and/or defense, and thus contain a complex mixture of different components to support these efforts^2^. In order to gather a better understanding of the functional role of enzymes across spider venoms, we classified all identified enzymes toward their putative chemical reaction, as well as venom- and or cellular function. We were able to assign 24 of 144 enzyme families to different venom functions and 17 to cellular functions. In addition, we further classified some enzyme families based on their catalyzed reaction or given their previously determined function (Supplementary Table 4)^10,34,41,56^.

The Delgado-Prudencio classification describes six known global venom functions based on physiological effects in relation to the enzymatic activities and their possible role in envenomation: toxic enzymes, prey pre-digestion enzymes, spreading factors, venom component activators, preservatives and multifunctional enzymes^39^. Toxic enzymes are one of the most relevant enzymatic venom compounds because they are directly related to the toxicity and physiological effects triggered by envenomation^39^. Enzymes with digestive functions are important for prey pre-digestion, whereas enzymes that compromise tissue integrity help to promote the distribution of other venom components^39^. Enzymes are also required to activate venom components by proteolysis or other forms of modification^39^. Enzymes with preservative functions help to eliminate reactive oxygen species (ROS), which are the intermediates or end products of cellular metabolism^57^. Venom components are susceptible to ROS, which results in a shorter half-life^58^. Many venom enzymes have multiple functions, and these have been assigned to a “multifunctional enzyme” category rather than specifying the individual functions^39^.

Oxidoreductases are required for toxin folding. Their diversity in spider venom indicates that the venom gland is highly active, producing many venom peptides/proteins that require assistance to adopt their correct tertiary structures^40^. This enzyme class is the third most common in our study. Some have been assigned a preservative function, while others are required to activate venom components. Two examples for the protection against oxidative stress are superoxide dismutases and peroxidases^39,59^.

Transferases modify other proteins by transferring functional groups. For example, kinases in endoparasite venom chemically modify proteins by adding phosphate groups that help to inhibit the host immune system^60^. We found many transferases, mostly kinases, but we were unable to assign specific venom functions. Because they contribute to the inhibition of the host immune system in endoparasites, they may act as defense toxins in spider venom by suppressing the prey’s immune system during envenomation^61^. Additionally, the Diniz classification assigned some kinases to cellular functions^40^, nucleoside-diphosphate kinases, for example, primarily participate in metabolic processes^41^. The injection of *Loxosceles intermedia* crude venom into rats increased the plasma levels of several transferases, and induced histopathological changes that indicated hepatic lesions^62^. Transferases may therefore play a role in the inhibition of the prey’s immune system, or may fulfill cellular functions beyond the function of venom, or may have a direct physiological effect on the prey.

Interestingly, the largest proportion of identified spider venom enzymes stemmed from hydrolases (Fig. 1). Those break down larger biomolecules into smaller units, e.g., for the digestive system^63^ or to facilitate the metabolism of extracellular matrix components^64–66^. Many of the biological functions associated with spider venom revolve around such catabolic effects, including pre-digestion, role as spreading factors or venom component activation. Therefore, it is relatively unsurprising that enzyme classes inherently involved in central venom functions are discovered at high abundance. Also, several of those hydrolase activities may serve multiple functions. For instance, the degradation of biomolecular components not only helps with pre-digestion but may also facilitate the diffusion/spreading of other venom components, which explains why many of these enzymes have been assigned as pre-digestive and spreading factors. The occurrence of hydrolases as major enzymes in spider venom also follows a general pattern. Many major compounds in diverse animal venoms are hydrolases, for example, metalloproteases or serine proteases, which participate in the harmful effects of snake (Elapidae, Viperidae) and spider (Sicariidae) venoms^67,68^. Phospholipase A2 and acetylcholinesterase are hydrolases that are well-known as toxic venom enzymes. Acetylcholinesterases cause neurological alterations, and are responsible for the flaccid paralysis triggered by snake venom^69^. Protein disulfide isomerases, protein tyrosine phosphatases and trypsin have been associated to cellular functions by supporting the protein folding or post-translation modifying venom components^41^.

As venom production is an energetically expensive process, venomous animals adjust venom expenditure in a context-dependent manner (“venom-metering”)^11,70,71^. Spiders use their venom mostly for predation or defense and are well-known to apply venom-metering and to target specific sites for venom injection^11,40,70–73^. One possibility to supplement the frugal use of venom is to utilize selected venom components for multiple functions and examples of venom enzymes serving multiple functions have been documented. For instance, several enzymes not only occur in the venom but also in the digestive fluids of venomous and non-venomous spiders and may serve a dual function^63,74–79^. Digestive fluids contain a mixture of hydrolases such as lipases, proteases, carbohydrases and nucleases^77–79^. The study of the extra-oral digestion by the spider *Nephilingis cruentata* for instance revealed the presence of astacins, trypsins, chitinases and others^79^. While the biological activity of these is limited to extraintestinal digestion, their counterparts within the venom system may be involved in intoxication as predicted by the dual prey inactivation strategy *sensu* Kuhn-Nentwig et al.^23^, but also serve pre-digestion^38,80^. Other simultaneously facilitated activities may include precursor activation or a functionality as spreading factors for neurotoxins^38,80^. Hyaluronidases for example are commonly reported enzymes present in spider venom and are proposed to act as spreading factors by degrading extracellular matrix components such as hyaluronan. As hyaluronan is found only in vertebrates and bacteria^81^, it is unlikely that hyaluronidases may play a key role in the predation of invertebrates. Therefore spider venom hyaluronidases are probably primarily employed as defensive weapons against vertebrate predators, in which they act as a spreading factor and as an allergen^82^. Likewise, PLDs are important toxic enzymes that usually have hydrolase activity but sometimes also facilitate transphosphatidylation^83^. Although the biological significance of this remains unresolved, this implies that at least some PLDs may serve multiple biological functions. This question requires more scientific attention and should be addressed in future studies. A last example for potentially multifunctional components are lysozymes, which are known to be defensive, antibiotic compounds that catalyze the hydrolysis of glycosidic *β*-(1,4) bonds in peptidoglycans and chitodextrins and exert antioxidant activity^38,84^. However, some lysozymes have been shown to further display isopeptidase- and chitinase activity^85^. Their biological function therefore may vary depending on the type and characteristics of the enzyme. The biological activity of spider venom lysozymes, similarly to most spider venom enzymes, needs to be established. However, based on the known activity spectrum and considering their absence from digestive spider secretions, they may be involved in defense and precursor activation^74,78,79^.

Besides the biological repercussions discussed above, our increased inventory of spider venom enzymes also opens so far untouched avenues for translational research.

In the past, spider venom research was primarily pharmacology-driven^51^. Thanks to the neurotoxic nature of many components, they were considered a prolific source of drug leads targeting diseases of the central nervous system (e.g. epilepsy, pain or stroke)^13,16,19,86^. As spider venom neurotoxins evolved to overpower insect prey and owed to their biodegradability, they were further heavily investigated for their potential to serve as eco-friendly insecticides^87,88^. A last angle taken by spider venom biodiscovery was the anti-infective nature of some venom components, particularly of linear venom peptides from RTA-clade spiders^89–91^. However, an entirely new area of biodiscovery may emerge through spider venom enzymes.

Enzymes are of utmost biotechnological importance as they facilitate a range of chemical reactions that are employed in industrial processes and every-day goods with efficiency^92,93^. Further, they perform these at mild reaction conditions, excellent yields with low by product release and are biodegradable^92^. As such, enzymes represent attractive components for the bioeconomy that can be used in a range of applications and have a particular value for a sustainable transition of industrial production. A range of spider venom enzymes belongs to classes that are particularly attractive, including chitinases (side-stream reduction in insect protein production and aquaculture)^94^, proteases (waste reduction and detergents) or oxidoreductases (targeted chemical synthesis)^92–94^. Unfortunately, this breadth of potentially attractive biomolecules remains almost completely unstudied as of yet.

We present the first systematic and comprehensive analysis of enzyme diversity in spider venoms based on database searches and manually screening previously published proteo-transcriptomic datasets. Enzymes have, historically, been considered minor and biologically less important components of spider venoms. However, a growing body of recent works challenged this perception. These studies suggested that spider venom enzymes may be more diverse and more biologically significant, than previously acknowledged. Here, we provide additional evidence for the key role of spider venom enzymes by unveiling an unprecedented chemical diversity, which is comparable to that of neurotoxins. Linked to that hitherto unknown molecular space, we unfolded an array of key biological functions in which these enzymes may be involved. We assembled 144 enzyme families and proteins with putative enzymatic activities representing 17 spider families. Most of the enzymes were hydrolases, oxidoreductases or translocases. We were able to assign 44 enzyme families to different venom functions or associated cellular functions. The venom functions included venom toxicity, spreading factors, venom activation and preservation, or prey pre-digestion, whereas the cellular functions included metabolism and the formation of cellular components. Most of the described enzymes were not previously associated with specific catalytic activities or venom functions. While theoretically annotating enzymes to specific functions is an important first step, extensive future experiments are required to experimentally validate these functions, which then helps in further improving theoretical annotations.

## Supplementary information

supplementary_table_1_Dresler_et_al_2024_npjbiodiversity_resubmission

supplementary_table_2_Dresler_et_al_2024_npjbiodiversity_resubmission

supplementary_table_3_Dresler_et_al_2024_npjbiodiversity_resubmission

supplementary_table_4_Dresler_et_al_2024_npjbiodiversity_resubmission_V2

## Data Availability

All data generated or analyzed during this study are included in this article or provided in supplementary information files.
